# TGF-β regulated Tim-3 sustains macrophage phagocytic function and confers protection in *Plasmodium yoelii* NSM-infected mice

**DOI:** 10.1186/s13071-026-07287-3

**Published:** 2026-02-27

**Authors:** Xiongyu Xie, Guikuan Liang, Mingjie Chen, Lixin Luo, Haiwen Yuan, Shenao Chen, Keyu Lu, Wenbo Peng, Long Xu, Hongyan Xie, Lu Li, Shan Zhao, Haixia Wei, Xingfei Pan, Jun Huang

**Affiliations:** 1 Department of Infectious Diseases, Key Laboratory for Major Obstetric Diseases of Guangdong Province, The Third Affiliated Hospital, Guangzhou Medical University, Guangzhou, China; 2 Key Laboratory of Immunology, Sino-French Hoffmann Institute, School of Basic Medical Sciences, Guangzhou Medical University, Guangzhou, China; 3 Department of Laboratory Medicine, The Sixth Affiliated Hospital of Guangzhou Medical University, Qingyuan People’s Hospital, Qingyuan, China; 4 Guangdong Provincial Key Laboratory of Allergy and Clinical Immunology, The Second Affiliated Hospital, Guangdong Provincial Key Laboratory of Allergy and Clinical Immunology, Guangzhou Medical University, Guangzhou, China

**Keywords:** Malaria, Tim-3, Macrophage, TGF-β, Phagocytosis

## Abstract

**Background:**

T-cell immunoglobulin and mucin domain 3 (Tim-3) is a critical immune checkpoint, yet its role in regulating macrophage function during malaria infection remains poorly understood.

**Methods:**

We established a *Plasmodium yoelii* NSM murine model, in vitro co-culture systems, and comprehensive techniques including scRNA-seq, flow cytometry, and functional assays to investigate Tim-3 expression on splenic macrophages and its immunoregulatory impact.

**Results:**

We observed a significant infection-induced downregulation of Tim-3 on splenic macrophages. Transcriptomic profiling revealed that Tim-3^+^ macrophages exhibited enhanced antigen presentation and a proinflammatory phenotype characterized by elevated reactive oxygen species (ROS) and proinflammatory cytokine production. Blockade of Tim-3 in vivo exacerbated disease severity, increased parasitemia, and impaired macrophage phagocytic capacity, without directly affecting T-cell responses. Mechanistically, we identified transforming growth factor-beta (TGF-β) as a key upstream regulator of Tim-3 expression, as TGF-β signaling was suppressed during infection, and its stimulation or inhibition correspondingly upregulated or downregulated Tim-3. Furthermore, TGF-β-induced Tim-3 upregulation potentiated macrophage phagocytosis of infected red blood cells (iRBCs) and conferred protection against iRBC-induced cell death.

**Conclusions:**

Our results reveal a novel protective TGF-β–Tim-3 axis that maintains the phagocytic function of macrophages and immune homeostasis in *Plasmodium yoelii* NSM infection. These findings highlight Tim-3 on macrophages as a potential therapeutic target for modulating host defense against malaria infection.

**Graphical Abstract:**

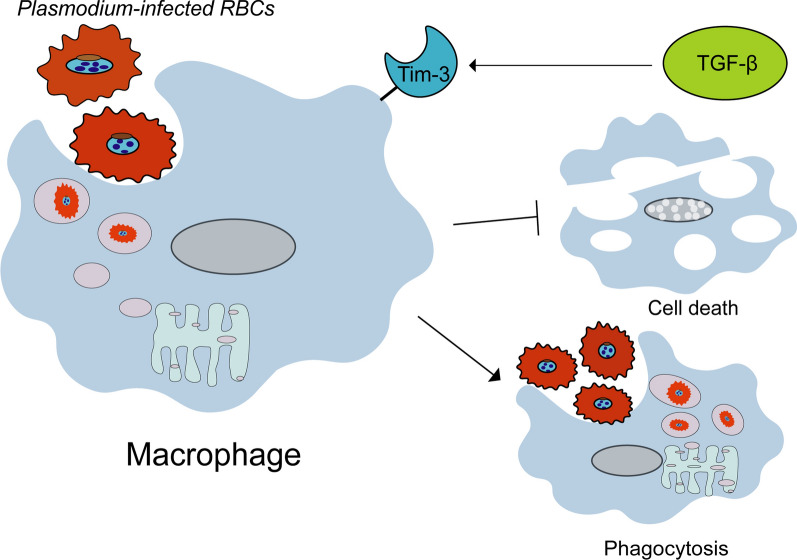

**Supplementary Information:**

The online version contains supplementary material available at 10.1186/s13071-026-07287-3.

## Background

Malaria, a parasitic disease caused by *Plasmodium* infection, represents the most lethal global parasitic disorder [1–3]. Malaria infections are categorized primarily into extracellular and intracellular stages, with the latter manifesting the most pronounced clinical symptoms—including fever, anemia, and splenomegaly. This infection triggers a robust innate immune response, which not only initiates protective acquired immunity, but also exerts direct antiparasitic effects [4, 5]. Such immunity is predominantly mediated by monocytes and macrophages, the latter critically mediating phagocytosis and antigen presentation against *Plasmodium* infection [6].

In recent years, Tim-3 has emerged as a critical class of immunoregulatory receptors [7]. Initially characterized as primarily expressed on T cells, Tim-3 is now established to be present on diverse myeloid immune cells, including macrophages and dendritic cells (DCs). Functionally, in Alzheimer’s disease, Tim-3 differentially regulates pathology via opposing effects on dendritic cells and microglia [8, 9]. In contrast, during malaria infection, Tim-3 upregulation exhibits a dual role: it acts as an activation marker on T cells associated with enhanced cytolytic function in the acute phase [10], but transitions to a key regulator of T cell exhaustion in chronic or severe infection [11]. Furthermore, emerging evidence implicates the Tim-3 immune checkpoint in defense against parasitic infections [12]. *Leishmania donovani*-induced Tim-3-mediated suppression of DCs critically compromises anti-leishmanial immunity [13]. Correspondingly, Tim-3 inhibition on immune cells enhances systemic immunity to combat malaria [14, 15].

In the early stage of *Plasmodium* infection, macrophages recognize the *Plasmodium*-related molecular patterns through pattern recognition receptors (such as Toll-like receptors) and promptly initiate the immune response. Studies have shown that macrophages can effectively phagocytose and eliminate infected red blood cells (iRBCs) and malaria pigment (hemozoin) caused by *Plasmodium* [16]. In the later stage of malaria infection, macrophages are overly activated, producing a large amount of proinflammatory factors such as tumor necrosis factor-alpha (TNF-α) and interleukin (IL)-6. Although it can help eliminate the parasites, it is also a key reason for severe malaria symptoms such as fever and hypoglycemia, as well as fatal complications such as cerebral malaria [17, 18]. In most other chronic inflammatory conditions (such as sepsis and colitis) and tumors (such as colon cancer and glioma), the activation of Tim-3 tends to inhibit the proinflammatory (M1) function of macrophages and promote their antiinflammatory and repair (M2) phenotype [19, 20]. However, in acute injuries such as the myocardial infarction model, the binding of Tim-3 to its ligand HMGB-1 may also exacerbate the inflammatory response [21]. Given the dual role of Tim3 macrophages in various diseases and stages, it suggests that Tim3 macrophages may play a crucial immunomodulatory role in the process of malaria infection.

Here we investigated the role of Tim-3 in splenic macrophages in the context of *P. yoelii* NSM infection. Tim-3 expression in splenic macrophages was markedly downregulated post-infection, and we demonstrated TGF-β-mediated regulation of this process. Antibody-mediated blockade of Tim-3 in splenic macrophages exacerbated infection progression in a murine malaria model, evidenced by significantly elevated parasitemia and accelerated weight loss. Moreover, TGF-β-induced Tim-3⁺ macrophages exhibited enhanced phagocytic capacity and reduced mortality rates following iRBC co-culture. Therefore, these findings delineate checkpoint molecule dynamics in splenic macrophages post-infection and nominate Tim-3 as a promising immunotherapeutic target against malaria.

## Methods

### Experimental animals and infection model

Female C57BL/6 mice (6–8 weeks old) were purchased from Guangzhou Zhiyuan Biological Co., Ltd and housed under specific-pathogen-free (SPF) conditions in the Experimental Animal Center of Guangzhou Medical University. Mice were maintained on a 12 h light/dark cycle with free access to food and water. The nonlethal *yoelii* NSM strain (MRA-427, contributed by D. Walliker, MR4, ATCC^®^ Manassas, VA) was used for infection. Mice were intraperitoneally (i.p.) injected with 1 × 10^6^ *P. yoelii*-infected red blood cells (iRBCs) suspended in 200 µL sterile PBS.

### Animal welfare and monitoring

All animal experiments were conducted in accordance with protocols approved by the Institutional Animal Care and Use Committee of Guangzhou Medical University (GY2020-134). Mice were monitored at least twice daily for predefined clinical signs of distress. Strictly, predefined humane endpoints were enforced, upon which mice were promptly and humanely euthanized under anesthesia. This specific study protocol was conducted within the validity period of the aforementioned ethical approval, and all procedures were performed in strict adherence to the approved guidelines throughout the study duration.

### Reagents and antibodies

The following fluorescently labeled antibodies were purchased from Biolegend (USA): FITC anti-mouse CD3 (Cat. No. 100306), Alexa Fluor 700 anti-mouse CD45 (Cat. No. 103128), Percp-cy5.5 anti-mouse CD19 (Cat. No. 152406), APC anti-mouse interferon‐gamma (IFN-γ) (Cat. No. 505810), APC anti-mouse IL-6 (Cat. No. 504508), APC anti-mouse IL-10 (Cat. No. 505010), PB450 anti-mouse TGF-β (Cat. No. 141408), APC-cy7 anti-mouse/human CD11b (Cat. No. 101226), PE/Cyanine7 anti-mouse F4/80 (Cat. No. 123114), APC anti-mouse CD11b (Cat. No. 101211), APC anti-mouse CD69 (Cat. No. 104514), and FITC anti-mouse MHC-II (Cat. No. 116406). The RPMI 1640 (REF C11875500BT 500 mL), collagenase IV (Cat. No. 17104019), and fetal bovine serum (FBS, Cat. No. FS301-02), penicillin and streptomycin (100×, 5000 U/mL, Cat. No. REF 15070-063 100 mL) were purchased from Gibco USA. The DNase I (Cat. No. AMPD1), Ionomycin (Cat. No. I3909), myristoyl phorbol ethyl ester (PMA, Cat. No. P1585), were purchased from Sigma USA. Brefeldin A (Cat. No. B5936) and Ionomycin (Cat. No. I3909) were purchased from Sigma. The cell fixation rupture solution kit solution (Cat. No. 554714) was purchased from BD USA.

### Cell culture and treatment

The murine macrophage cell line RAW264.7 was obtained from the American Type Culture Collection (ATCC). RAW264.7 cells were cultured in complete DMEM medium supplemented with 10% fetal bovine serum (FBS). The cells were incubated at 37 °C in a humidified atmosphere containing 5% CO_2_. For experiments, subsequently, cells were co-cultured with iRBCs for 5 h to assess changes in Tim-3 expression and for 12 h to measure phagocytosis.

### Isolation of spleen lymphocytes

Spleens were aseptically harvested and mechanically dissociated through a 100-μm strainer using D-Hank’s solution. Cells were resuspended in complete RPMI 1640 medium (10% FBS, penicillin–streptomycin), and viability was assessed by hemocytometer counting.

### Preparation of infected red blood cells

Infected red blood cells (iRBCs) were collected from the orbital sinus of infected mice into EDTA-K2-coated tubes. After plasma removal, a discontinuous Percoll gradient (70% Percoll at the bottom, overlaid with 50% Percoll) was prepared in a centrifuge tube. The resuspended iRBCs were layered on top and centrifuged at 300× *g* for 30 min. iRBCs were collected from the 70%/50% Percoll interface and resuspended in PBS.

### RNA extraction and RT-qPCR

Total RNA was extracted from splenocyte suspensions using SteadyPure Universal RNA Extraction Kit II (Catalog No. Q711-02). cDNA was synthesized from total RNA using Fast first-Strand cDNA Synthesis Mix (Catalog No. 500-101). For this reaction, 1 μg of RNA was used. Quantitative real-time PCR (qRT-PCR) was performed using ChamQ Universal SYBR qPCR Master Mix (Catalog No. Q711-02) and the CFX96 Real-Time PCR Detection System. Data were normalized using β-actin and target gene-specific primers.

### Cell surface staining

Cells were washed twice with PBS, blocked with 1% BSA for 30 min, and stained with fluorochrome-conjugated surface antibodies for 30 min at 4 °C in the dark. Flow cytometry was performed on a Beckman Coulter instrument, and data were analyzed using CytoExpert 2.5 software.

### Intracellular cytokines and transcriptional factors staining

Splenocytes were stimulated with PMA (20 ng/mL, Cat. No. P1585) and ionomycin (1 μg/mL, Cat. NoI3909) for 6 h at 37 °C with 5% CO₂, with Brefeldin A (10 μg/mL, Cat. No. B5936) added for the final 4 h. After surface staining, cells were fixed, permeabilized, and intracellularly stained with cytokine-specific antibodies in saponin-containing buffer. Flow cytometric analysis was performed using a Beckman Coulter instrument and CytoExpert 2.5 software.

### Magnetic bead sorting

Mouse F4/80^+^ macrophages were isolated from mouse splenocytes using the EasySep™ Mouse F4/80 Positive Selection Kit (STEMCELL Technologies, Catalog No. 100-0659). The kit includes the following components: EasySep™ Mouse F4/80 Positive Selection Component A (0.3 mL), Component B (0.6 mL), EasySep™ Dextran RapidSpheres™ 50100 (2 × 1 mL), and Mouse FcR PolyBlock (1.2 mL, Catalog No. 300-0902).

### Measurement of phagocytic function

CFSE-labeled iRBCs were prepared by resuspending infected red blood cells at 5 × 10^6^ cells/mL in pre-warmed PBS containing 0.1% BSA. CFSE (Catalog No. C34554) was added at a final concentration of 5 μM and incubated for 20 min at 37 °C in the dark. The reaction was quenched with five volumes of complete medium (RPMI‑1640 + 10% FBS) at 4 °C, followed by three washes with complete medium (600× *g*, 5 min each). Labeled iRBCs were then co‑cultured with macrophages for 12 h, and macrophage‑associated CFSE fluorescence was analyzed by flow cytometry.

### *Invivo* and *in vitro* antibody and inhibitor treatments

For *in vivo* studies, *P. yoelii*-infected mice were treated via intraperitoneal injection on days 4 and 9 post-infection. For checkpoint blockade, mice received 200 µg of anti-mouse TIM-3 blocking antibody (BioXCell, Catalog No. BE0115) diluted in sterile PBS. For TGF-β signaling inhibition, mice were administered galunisertib (100 µg/kg; MCE, Catalog No. LY2157299). Galunisertib was prepared from a 10 mM stock solution in DMSO and diluted in sterile PBS to a final concentration of 5% DMSO. Control mice received an equivalent volume of the corresponding vehicle (PBS or 5% DMSO in PBS, respectively). For *in vitro* experiments, RAW264.7 macrophages were cultured in the presence of anti-TIM-3 antibody (10 µg/mL), or galunisertib (10 µM) for 12 h. Control cells were treated with the vehicle solvent alone. 

### Single cell sequencing reanalysis

In this study, splenic immune cells from normal C57BL/6 mice and *Plasmodium yoelii* NSM-infected C57BL/6 mice were subjected to single-cell capture and cDNA library construction using the 10× Genomics Chromium platform, followed by sequencing on an Illumina NovaSeq 6000 platform. The raw FASTQ files were obtained from the NCBI Gene Expression Omnibus (GEO) database under accession numbers SRR22462455, SRR22462456, and SRR22476069 (representing a total of 21,508 cells). Initial data processing, including sample demultiplexing, barcode processing, and single-cell 3′ gene counting, was performed using the Cell Ranger pipeline (version 3.1.0; 10× Genomics). The scRNA-seq reads were aligned to the Ensembl *Mus musculus* reference genome (GRCm38). All subsequent bioinformatic analyses were conducted within the R environment. Quality control filtering, data normalization, and dimensionality reduction were performed using Seurat (v4.3.0). Cell clusters were identified by constructing a shared nearest neighbor (SNN) graph on the basis of principal components and applying a graph-based clustering algorithm, with the results visualized using uniform manifold approximation and projection (UMAP). The FindAllMarkers function (employing the Wilcoxon rank-sum test) was utilized to identify cluster-specific marker genes, enabling the biological annotation of each cluster on the basis of established canonical immune cell markers. Differential gene expression analysis between conditions or clusters was carried out using the FindMarkers function (significance threshold: *P* value <0.01, |log_2_ fold change| > 0.58). Gene Ontology (GO) Biological Process and Kyoto Encyclopedia of Genes and Genomes (KEGG) pathway enrichment analyses were then performed on the differentially expressed genes using clusterProfiler (v4.6.2), applying both stringent (*P* < 0.01, |log_2_ fold change| > 0.58) and liberal (*P* < 0.1, |log_2_ fold change| > 0.25) significance thresholds. The activity of predefined gene sets (referenced in Supplementary Table 1) was scored using the AUCell algorithm (v1.12.0). Data visualization was primarily accomplished using the ggplot2 package (v3.3.5).

### RNAseq analysis

Differential gene expression analysis was performed using DESeq2, with significantly differentially expressed genes (DEGs) defined as those with an adjusted *P*-value (*P* adj < 0.01, |log_2_ fold change| > 0.58). GO biological process and KEGG pathway enrichment analyses were conducted on the DEGs using clusterProfiler (v4.0.5) with the Mus musculus annotation database (org.Mm.eg.db). Statistical significance was set at adjusted *P* value and *q*-value < 0.05 (BH method).

### Quantification and statistical analysis

Flow cytometry data were analyzed with the use of CytoExpert (version 2.5) and FlowJo (version 10.8.1). Descriptive analysis and statistical testing of flow cytometry and histology data were performed using Prism (GraphPad version 9.5.1). Multivariate analyses were performed using one-way analysis of variance (ANOVA) and two-tailed unpaired *t* test in Prism.

## Results

### Downregulation of Tim-3 on splenic macrophages during *Plasmodium yoelii* NSM infection in mice

**Figure 1:**
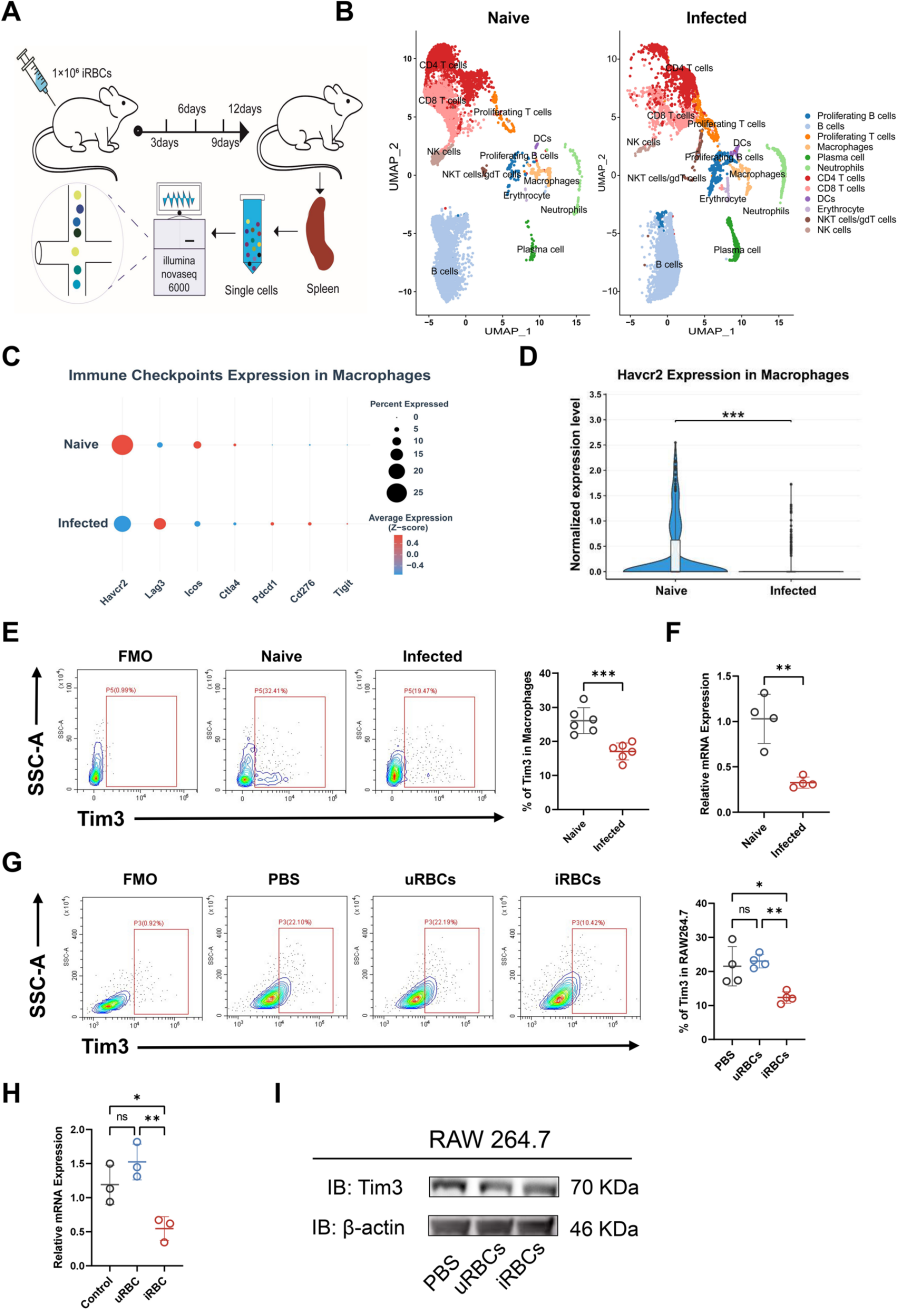
Tim-3 downregulation on splenic macrophages in *P. yoelii* NSM-infected mice. **A** C57BL/6 mice were infected with 1 × 10^6^ *P. yoelii* NSM parasites by intraperitoneal injection, and 12 days after the infection, spleen lymphocytes were collected for single-cell sequencing. **B** UMAP visualization of immune cell subsets in the spleen. **C** Violin plot depicting immune-checkpoint expression dynamics in splenic macrophage populations. **D** Violin plot demonstrates Havcr2 expression in splenic macrophages at the single-cell level. **E** Tim-3 expression levels of splenic macrophages in naïve and infected groups. **F** mRNA expression level of *Havcr2* in macrophages. **G** Tim-3 expression in RAW264.7 cells co-cultured with PBS, uRBCs, and iRBCs. **H** mRNA expression level of *Havcr2* in RAW264.7. **I** Western blot analysis of Tim-3 protein expression. Data are mean ± SEM from three independent experiments (*n* = 3–6 per group). *ns* not significant, *P* > 0.05, * *P* < 0.05, ** *P* < 0.01, *** *P* < 0.001; **D**–**F** two-tailed unpaired *t* test; **G**,**H** one-way ANOVA

As an immune checkpoint, Tim-3 is widely expressed on immune cells including T cells, macrophages, NK cells and so on [7]. To investigate the possible alteration of Tim-3 in malaria infection, Tim-3 levels in splenic macrophage of wild-type naïve and infected mice were assessed by scRNA-seq, qPCR, flow cytometry and western blot. scRNA-seq was performed on splenic immune cells to map the cellular landscape before and after malaria infection (Fig. 1A, B). Through average expression of key immune-checkpoint factors, including *Havcr2*, *Lag3*, *Icos*, *Ctla4*, *Pdcd1*, *Cd276*, and *Tigit*, we observed a significant downregulation of *Havcr2* specifically in splenic macrophages post-infection (Fig. 1C, D). Splenic macrophages were identified and isolated based on their co-expression of CD45⁺, CD11b⁺ and F4/80⁺ surface markers (Supplementary Fig. 1A). Similarly, a decrease of Tim-3 levels in splenic macrophages by flow cytometry after malaria infection in comparison to those uninfected mice (Fig. 1E). qPCR analysis demonstrated the mRNA expression of Tim-3 was significantly downregulated in splenic macrophages post-infection (Fig. 1F; Supplementary Fig. 1B). In addition, to further confirm the downregulation of Tim-3 after *P. yoelii* NSM infection, we established a co-cultured model in which RAW264.7 cells were cultured with uRBCs and iRBCs for 5 h in vitro. Our results showed that Tim-3 levels on RAW264.7 cells was marked reduced after phagocytosis of iRBCs, as shown by both protein and mRNA (Fig. 1G, H). Notably, the extent of Tim-3 downregulation was more pronounced in cells co-cultured with iRBCs than those exposed to uRBCs (Fig. 1I). Therefore, these results demonstrate that Tim-3 expression on macrophages is robustly downregulated after *P. yoelii* NSM infection. This suggests that Tim-3 might plays a pivotal role in modulating macrophage function during malaria infection.

### Proinflammatory polarization of splenic Tim-3^+^ macrophages during *Plasmodium yoelii* NSM infection

**Figure 2:**
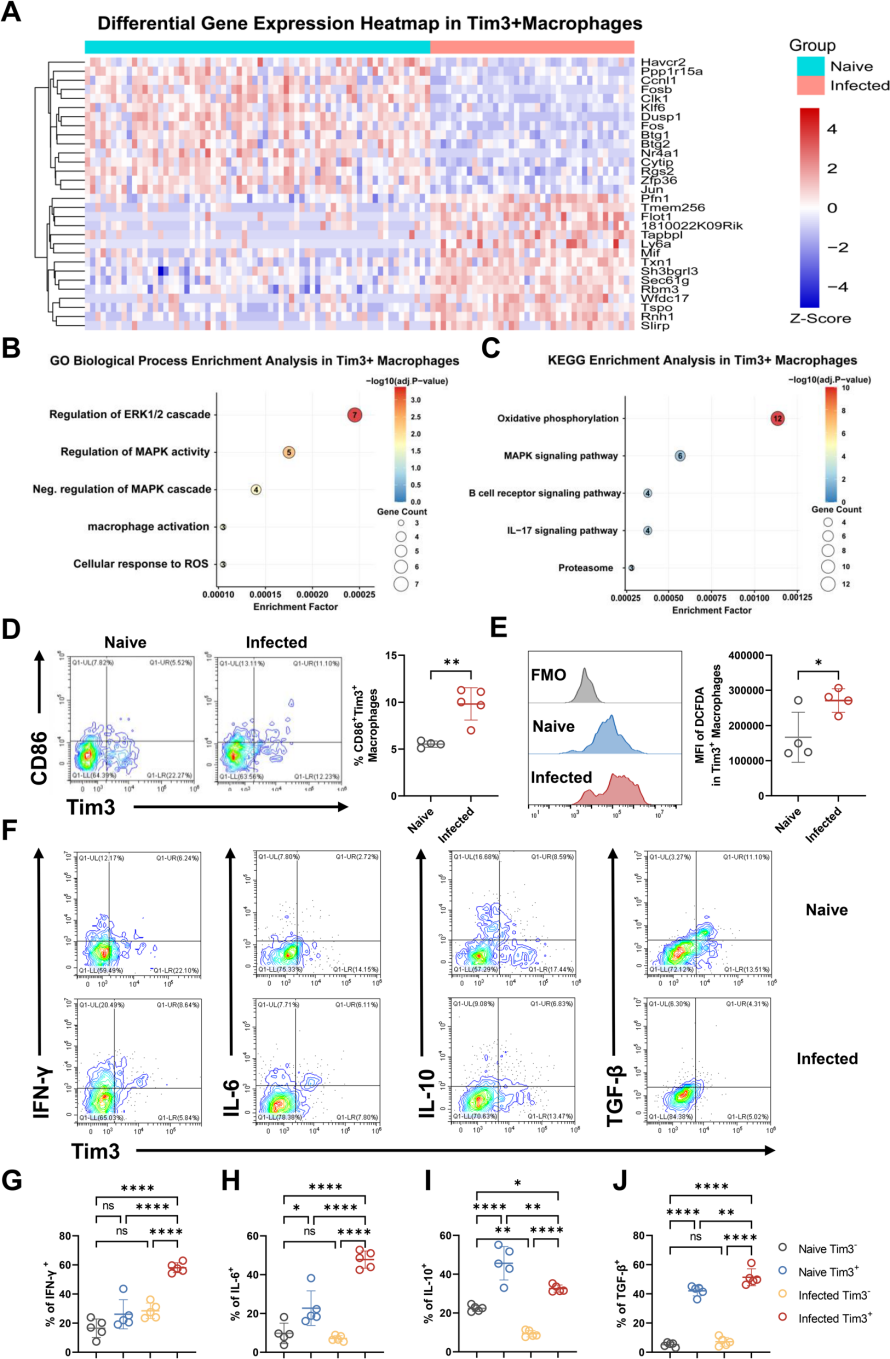
Proinflammatory polarization of splenic Tim-3^+^ macrophages during *P. yoelii* NSM infection. **A** Heatmap of differentially expressed genes (DEGs) between naïve and infected Tim-3^+^ macrophages. **B**,**C** Enriched gene ontology (GO) terms and KEGG pathways in Tim-3^+^ macrophages post-infection. **D** CD86 expression on splenic Tim-3^+^ macrophages. **E** ROS accumulation in splenic Tim-3^+^ macrophages. **F** Flow cytometry gating strategy for cytokine (IFN-γ, IL-6, IL-10, TGF-β) expression in splenic macrophages. **G**–**J** Secretion levels of cytokine (IFN-γ, IL-6, IL-10, TGF-β) in splenic macrophages. Data are presented as mean ± SEM from three independent experiments (*n* = 4–5 per group). *ns* not significant, *P* > 0.05, * *P* < 0.05, ** *P* < 0.01, **** *P* < 0.0001; **D**,**E**,**G**–**J** two-tailed unpaired *t* test

To investigate the role of Tim-3-expressing macrophages in malaria, we began by comparing the transcriptional profiles of sorted Tim-3^+^ and Tim-3^−^ splenic macrophages. Key antigen-presentation molecules, including *Ciita*, *Cd74*, *H2-Aa*, *H2-Ab1*, *H2-Eb1*, and *H2-DMb1*, were significantly upregulated in Tim-3^+^ splenic macrophages, suggesting an enhanced antigen-presenting capacity (Supplementary Fig. 2A). Consistent with this, employing a liberal threshold for gene set variation analysis (GSVA) in conjunction with stringent GO/KEGG filters identified immune response and antigen presentation pathways were significantly enriched in Tim-3^+^ versus Tim-3^−^ macrophages (Supplementary Fig. 2B). To further delineate the infection-specific impact, we next compared the gene expression profiles of Tim-3^+^ macrophages from naïve and infected mice. Key immune response-inflammatory molecules, including *Mif*, *Dusp1*, *Fos*, *Jun*, *Zfp36*, and *Nr4a1*, exhibit significant alterations on Tim3^+^ macrophages post-infection, indicating that these Tim3^+^ macrophages are in a highly stressed state and possess significant immunoregulatory functions (Fig. 2A). GO biological process (BP) and KEGG pathway enrichment analyses showed significant enrichment in processes related to reactive oxygen species (ROS) production and the response to oxidative stress, which is consistent with the highly stressed state of this cell population (Fig. 2B, C). Additionally, infected mice exhibited a significantly higher percentage of CD86^+^Tim-3^+^ splenic macrophages than naïve mice (Fig. 2D), suggesting a potential shift toward a proinflammatory polarization. Tim-3^+^ splenic macrophages displayed elevated levels of reactive oxygen species (ROS) following *P. yoelii NSM* infection (Fig. 2E). Interestingly, infection with Tim-3^+^ macrophages derived from mice exhibited a trend toward increased production of proinflammatory cytokines (such as IFN-γ and IL-6), while the secretion of antiinflammatory mediators (including IL-10) was significantly reduced (Fig. 2F, J). Collectively, these findings indeed highlight that Tim-3 plays a pivotal role in modulating the immune response by promoting a proinflammatory phenotype in splenic macrophages during malaria infection, thereby contributing to the maintenance of immune homeostasis.

### Blocking Tim-3 exacerbated the severity of *Plasmodium yoelii* NSM infection in mice

**Figure 3:**
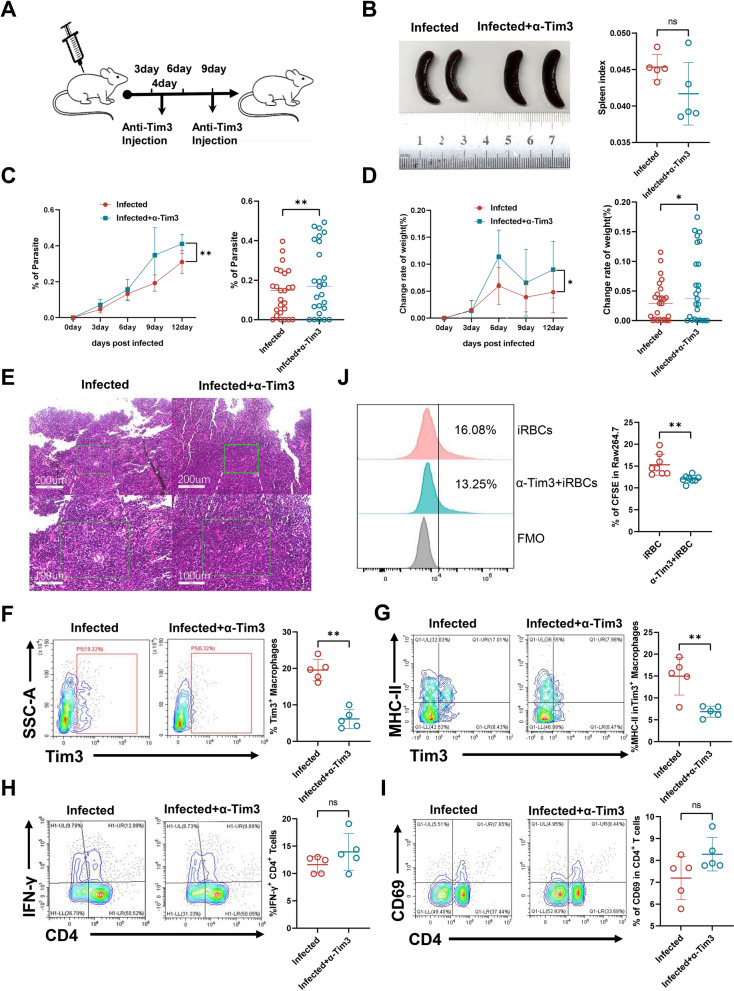
Inhibition of Tim-3 expression in splenic macrophages exacerbated *P. yoelii* NSM infection. **A** Mice received intraperitoneal injections of anti-Tim-3 blocking antibody (BE0115, 200 μg/mouse) on days 4 and 9 post-infection. **B** Spleen morphology of infected mice after injection. **C** The line graph illustrated the protozoan rate after Tim-3 blockade in C57BL/6 mice. **D** The line graph illustrated the rate of body weight change after Tim-3 blockade in C57BL/6 mice. **E** Histopathological analysis of spleen sections from infected mice. **F** Expression of Tim-3 on splenic macrophages in infected and anti-Tim-3 groups. **G** Expression of MHC-II on Tim-3^+^ macrophages. **H** Secretion levels of IFN-γ in CD4^+^ T cells. **I** Expression of CD69 in CD4^+^ T cells. Data are presented as mean ± SEM from three independent experiments (*n* = 5–25 per group). *ns* not significant, *P* > 0.05, * *P* < 0.05, ** *P* < 0.01; **B**–**D**,**F**–**J** two-tailed unpaired *t* test

Considering that Tim-3 exerts diverse functions in different immune cells and diseases [22–24], to investigate the role of Tim-3-expressing splenic macrophages in malaria, we administered anti-Tim-3 blocking antibody intraperitoneally on days 4 and 9 post-infection, aiming to suppress Tim-3 expression in splenic macrophages and evaluate their impact on the progression of malaria (Fig. 3A). Despite no significant differences in spleen morphology between the anti-Tim-3-treated and untreated infected mice (Fig. 3B), our findings revealed a marked exacerbation of disease severity in the anti-Tim-3-treated group. Blockade of Tim-3 exacerbated disease severity, leading to increased weight loss and elevated parasitemia compared with the control antibody-treated group (Fig. 3C, D). Histopathological analysis of splenic tissues further supported these observations, demonstrating enhanced inflammatory cell infiltration in the anti-Tim-3-treated mice, suggesting Tim-3 blockade that may promote *Plasmodium* infection (Fig. 3E). Furthermore, flow cytometry confirmed that anti-Tim-3 treatment significantly reduced Tim-3 expression on splenic macrophages, which validated the efficacy of the blockade (Fig. 3F). Tim-3 inhibition led to a concomitant reduction in MHC-II expression on Tim-3^+^ macrophages (Fig. 3G), suggesting impaired antigen-presenting capacity. Importantly, functional assays demonstrated that Tim-3 blockade markedly diminished the phagocytic capacity of macrophages toward CFSE-iRBCs, underscoring the essential role of Tim-3 in mediating macrophage-dependent parasite clearance (Fig. 3J; Supplementary Fig. 3A). Given the central role of macrophages in activating T-cell responses, we further assessed whether Tim-3 blockade influenced T cell activation or cytokine production. However, no significant differences were observed in the percentage of CD69^+^CD4^+^ T cells and IFN-γ^+^CD4^+^ T cells between the two groups (Fig. 3H, I). Collectively, these results demonstrated that Tim-3 expression on splenic macrophages is essential for mitigating disease severity during *Plasmodium* infection.

### TGF-β modulated Tim-3 expression on splenic macrophages in *Plasmodium yoelii* NSM-infected mice

**Figure 4:**
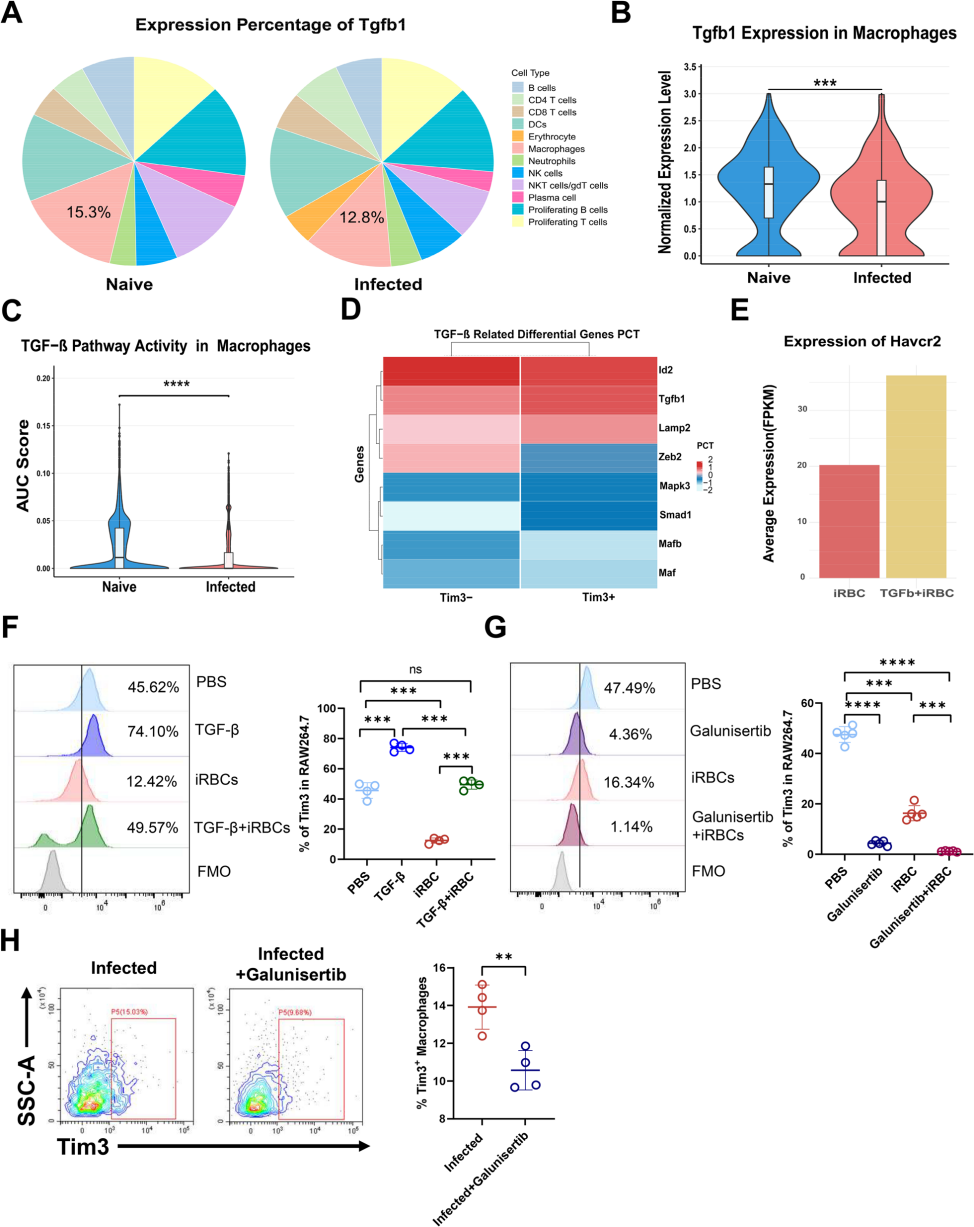
TGF-β modulates Tim-3 expression on splenic macrophages in mice of *P. yoelii* NSM infection. **A** The percentage of TGF-β in macrophages of naïve and infected groups. **B** Expression levels of TGF-β in macrophages. **C** AUCell score of the TGF-β pathway activity in macrophages. **D** Heatmap shows expression of TGF-β pathway genes in Tim-3^+^ and Tim-3^−^ macrophages. **E** Average expression (FPKM) of *Havcr2* on Raw264.7 after TGF-β co-culture. **F** Expression levels of Tim-3 after co-culture with TGF-β and iRBCs. **G** Expression levels of Tim-3 after co-culture with galunisertib and iRBCs. **H** The expression of Tim-3 in the infected and the infection-injected galunisertib groups. Data are mean ± SEM from three independent experiments (*n* = 4–5 per group). *ns* not significant, *P* > 0.05, ** *P* < 0.01, *** *P* < 0.001, **** *P* < 0.0001; **B**,**C**,**H** two-tailed unpaired *t* test; **F**,**G** one-way ANOVA

There are substantial reports suggesting that TGF-β plays a critical role in modulating immune homeostasis during malaria infection [25, 26], and its expression change closely parallels that of the Tim-3 molecule (Fig. 2F, J). This correlation prompted us to hypothesize that TGF-β may be responsible for inducing Tim-3 expression in macrophages following *Plasmodium* infection. Supporting this hypothesis, scRNA-seq analysis exhibited a marked decrease in TGF-β expression within splenic macrophages following infection (Fig. 4A, B). The AUCell algorithm assessed the activity of TGF-β pathway in macrophages from naïve and infected groups, revealing a significant decrease in TGF-β pathway activity following infection (Fig. 4C). Among TGF-β-associated genes, Tim-3^+^ macrophages demonstrated elevated *Tgfb1* expression, suggesting a potential regulatory mechanism wherein TGF-β modulates Tim-3 expression on macrophages (Fig. 4D). Consistent with this observation, TGF-β mediated pronounced Tim-3 upregulation in RAW264.7 macrophages under iRBC co-culture conditions (Fig. 4E). To functionally validate this relationship, we conducted co-culture experiments using macrophages and iRBCs, with or without TGF-β stimulation. We found that TGF-β significantly enhanced Tim-3 expression in RAW264.7 macrophages co-cultured with iRBCs (Fig. 4F). In contrast, pharmacological inhibition of TGF-β signaling using galunisertib, a TGF-β receptor inhibitor, led to a significant reduction in Tim-3 expression under the same co-culture conditions (Fig. 4G). Blockade of TGF-β also resulted in diminished Tim-3 expression on macrophages in vivo (Fig. 4H). Therefore, these findings provide strong evidence that TGF-β signaling pathway serves as a potent inducer of Tim-3 expression in macrophages during *Plasmodium* infection.

### Upregulation of Tim-3 expression enhanced the phagocytosis of iRBCs by Tim-3^+^ macrophages and reduced cell death

**Figure 5:**
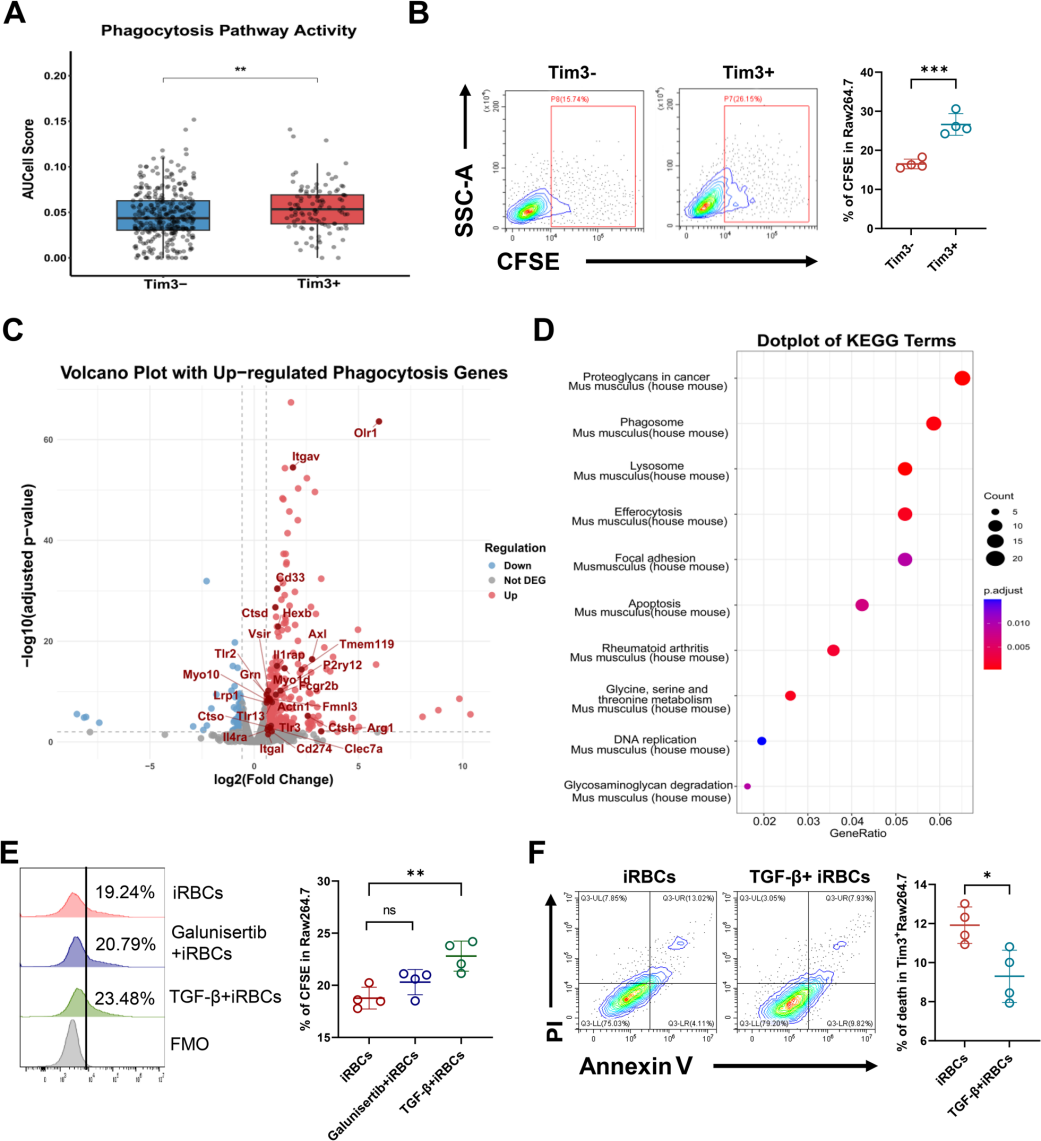
Tim-3^+^ macrophages exhibit enhanced phagocytic activity during *P. yoelii* NSM infection. **A** The box plot of the AUCell score was used to illustrate the phagocytic function of Tim-3^+^ and Tim-3^−^ spleen macrophages. **B** The phagocytic function of Tim-3^+^ and Tim-3^−^ macrophages after co-culture with CFSE-labeled iRBCs. **C** The volcano plot shows the expression of genes related to phagocytosis in RAW264.7 cells after co-culture with TGF-β and iRBCs. **D** The enriched bubble chart presents the top ten differential enrichment pathways. **E** The proportion of phagocytosis of CFSE-iRBCs after adding galunisertib and TGF-β in the infected condition. **F** The level of apoptosis induced by TGF-β in the infected condition. Data are mean ± SEM from three independent experiments (*n* = 4 per group). *ns* not significant, *P* > 0.05, * *P* < 0.05, ** *P* < 0.01, *** *P* < 0.001; **B**,**F** Two-tailed unpaired *t* test; **E** one-way ANOVA

As established, the potent phagocytic capacity of macrophages is crucial for the elimination of iRBCs [4]. Our findings in Fig. 2 further demonstrate that Tim-3^+^ macrophages play a critical role in conferring resistance to malaria. Therefore, it is essential to investigate the dynamics of Tim-3 expression on macrophages following *Plasmodium* infection and its functional implications for phagocytosis. scRNA-seq analysis revealed elevated the phagocytosis pathway activity in Tim-3^+^ macrophages compared with Tim-3^−^ subsets (Fig. 5A). To validate the link between Tim-3 expression and enhanced iRBC clearance, we established a co-culture system to evaluate macrophage function. Notably, Tim-3^+^ RAW264.7 cells exhibited significantly greater engulfment of CFSE-labeled iRBCs than their Tim-3^−^ counterparts during co-culture (Fig. 5B). Building on observations from Fig. 4, we modulated Tim-3 expression using TGF-β and its inhibitor to assess associated changes in phagocytic activity. mRNA-seq analysis identified significant upregulation of phagocytosis-related genes (*ItgaV*, *Itga1*, *Ctsd*, *Cd33*, *Tlr2*, *Tlr3*, *Cd274* and so on) following TGF-β and iRBCs co-cultured (Fig. 5C). Stimulation with TGF-β also enhanced CFSE-iRBC phagocytosis in RAW264.7 cells (Fig. 5E). Furthermore, KEGG enrichment analysis confirmed significant “phagosome,” “efferocytosis,” and “apoptosis” pathway activation (Fig. 5D; Supplementary Fig. 2D), indicating a high-phagocytosis and low-cell-death state in Tim-3-upregulated macrophages. This phenotype was reinforced by TGF-β-mediated amelioration of iRBC-induced cell death in Tim-3^+^ RAW264.7 cells (Fig. 5F). Collectively, these results suggest that Tim-3 upregulation promotes a pro-phagocytic phenotype, while suppressing cell death in macrophages.

## Discussion

Our findings reveal a significant reduction in splenic Tim-3*+* macrophages during blood-stage *P. yoelii* NSM infection (Fig. 1; Supplementary Fig. 3B). Contrary to the established association between Tim-3 and T cell exhaustion in chronic diseases and cancer [27, 28], Tim-3 expression on splenic macrophages in our model correlated with a proinflammatory activation phenotype rather than an inhibitory state (Fig. 2D–J). Mechanistically, we demonstrated that TGF-β signaling upregulates Tim-3 expression, which in turn enhances the phagocytic capacity of these macrophages against infected erythrocytes and promotes their survival (Figs. 4, 5). This suggests that, in the context of nonlethal malaria, Tim-3 may serve as a marker of cellular activation and contribute to a protective innate immune response. Further mechanistic insight reveals a TGF-β-regulated protective Tim-3 axis in macrophages, which operates in stark contrast to the canonical inhibitory function of Tim-3 in T cells [29].

As a key multifunctional regulator of myeloid cells, Tim-3 displays context-dependent effects. Its deficiency in dendritic cells inhibits tumor growth, whereas loss in CD8^+^ T cells shows negligible impact [30]. This functional duality is particularly evident in infectious diseases, extending beyond its role in tumors. During systemic bacterial infection such as sepsis, Tim-3 upregulation on monocytes/macrophages can act as a crucial negative feedback mechanism to restrain excessive inflammation and prevent host tissue damage, particularly by inhibiting the TLR4-NF-κB pathway [31]. Conversely, in intracellular bacterial infections such as Listeria monocytogenes infection, Tim-3 signaling in macrophages inhibits bacterial clearance by suppressing phagocytic and bactericidal activity [32]. This functional duality underscores Tim-3’s capacity to mediate divergent immune responses under distinct disease microenvironments [21, 30]. Therefore, the observed Tim-3 downregulation in splenic macrophages during *P. yoelii* infection does not contradict prior reports but rather highlights the functional plasticity and context specificity of Tim-3-mediated immunoregulation [14]. This understanding sets the stage for examining its specific, and perhaps unique, functions in malaria infection.

In malaria, activated myeloid cells—including monocytes and macrophages—control malaria parasites through phagocytosis, producing ROS and nitric oxide (NO), and secreting proinflammatory cytokines (e.g., TNF-α, IL-1β, IFN-γ). These effector mechanisms collectively suppress parasite replication. Concurrently, immune checkpoint molecules including TIM-3, PD-1, TIGIT, and LAG-3 are significantly upregulated in T cells across multiple organs and peripheral blood [33, 34]. Studies using *Plasmodium berghei* ANKA infection models demonstrate that inhibition of Tim-3 on immune cells reduces parasitemia and improves survival rates, suggesting its immunoregulatory role in suppressing excessive inflammation [15, 35]. In contrast, in our study, we reveal Tim-3 downregulation on splenic macrophages during *P. yoelii* infection (Fig. 1E). These Tim-3^+^ macrophages exhibit a proinflammatory splenic phenotype (Fig. 2), and their expression blockade exacerbates *P. yoelii* NSM infection severity in mice (Fig. 3C). TGF-β, a key immunomodulatory cytokine, regulates Tim-3 expression in DCs and macrophages across multiple disease contexts [8, 9, 36]. Our results demonstrate that TGF-β upregulates Tim-3 on macrophages expression, enhancing phagocytosis of iRBCs while attenuating cellular death (Fig. 4, 5). Given these findings, it is reasonable to conclude that TGF-β-mediated regulation promotes Tim-3 expression on macrophages, thereby enhancing resistance to malaria progression.

Although the nonlethal *P. yoelii* model recapitulates the key features of human nonsevere malaria, such as splenomegaly and parasitemia, and provides a clear time window for analysis, our study has limitations. Firstly, the applicability of our conclusions requires further validation. Given our exclusive focus on the nonlethal *P. yoelii* infection model, it remains unresolved whether Tim-3 exerts analogous functions in macrophages during lethal *Plasmodium* infections or other bacterial/viral contexts. Secondly, the translational relevance of the identified TGF-β/Tim-3 axis requires validation in human contexts. While our study delineates this macrophage-protective pathway in a murine model, future work is essential to verify its conservation and function in primary human macrophages in vitro and to assess its activity in clinical samples from patients with malaria. Furthermore, more direct genetic or pharmacological perturbation experiments in vivo would be needed to conclusively establish the causal role of this axis in shaping antimalarial immunity.

In this study, we show that Tim-3^+^ macrophages exhibit a proinflammatory phenotype characterized by enhanced production of ROS and proinflammatory cytokines. Following anti-Tim-3 antibody treatment in C57BL/6 mice, these macrophages mediate increased parasite burden and exacerbate weight loss. Transcriptomic analyses reveal that Tim-3^+^ macrophages upregulate pro-phagocytic pathways following *P. yoelii* infection to promote iRBC clearance. Our findings reveal that a balanced TGF-β/Tim-3 axis is critical for protective splenic macrophage function, thereby nominating Tim-3 as a therapeutic target to ameliorate malaria pathogenesis. These results establish a novel framework for understanding the spectrum of disease outcomes in human malaria.

## Conclusions

This study delineates a protective role for Tim-3 on splenic macrophages during nonlethal *P. yoelii* infection. Contrary to its canonical function in T-cell exhaustion, it was demonstrated that Tim-3 expression marks a proinflammatory and activated macrophage population, the maintenance of which is crucial for controlling parasitemia and pathogenesis in this study. Mechanistically, we identify TGF-β as a key upstream regulator of Tim-3, whose expression enhances the phagocytic clearance of iRBCs and promotes macrophage survival. These findings collectively reposition Tim-3 as a positive regulator of innate immunity in this specific context, highlighting its functional plasticity. However, the broader applicability and mechanistic depth of our conclusions warrant further investigation. Future studies should seek to validate whether Tim-3 exerts analogous functions in macrophages during lethal malaria strains or other infectious diseases. Moreover, the precise signaling pathways governing TGF-β-dependent *Havcr2* transcription and the downstream mechanisms by which Tim-3 transduces its pro-phagocytic and pro-survival signals remain to be fully elucidated. Addressing these questions through more direct in vivo functional assays will be essential to solidify our understanding and assess the therapeutic potential of targeting the macrophage Tim-3 pathway in infectious diseases.

## Supplementary Information

**Additional file 1.**

**Additional file 2. Supplementary Table 1.** The TGF-β-related and phagocytic function-related gene set.

**Additional file 3. Supplementary Table 2.** Significantly differentially expressed genes between Tim-3^+^ and Tim-3^−^ macrophages.

**Additional file 4. Supplementary Table 3.** The primers of target genes for the qPCR method.

## Data Availability

The datasets generated and/or analyzed during the current study and other materials used are available from the corresponding author on reasonable request.

## Abbreviations

NSM: Non-lethal Strain
CFSE: Carboxyfluorescein succinimidyl ester
