# Supplementary material for: TGF-β regulated Tim-3 sustains macrophage phagocytic function and confers protection in Plasmodium yoelii NSM-infected mice
Source: Parasit Vectors. 2026 Feb 27;19:144. doi: 10.1186/s13071-026-07287-3 (PMC13040716; doi:10.1186/s13071-026-07287-3)
Supplement: Supplementary file 1 — Additional file 1. [file 13071_2026_7287_MOESM1_ESM.docx]

**
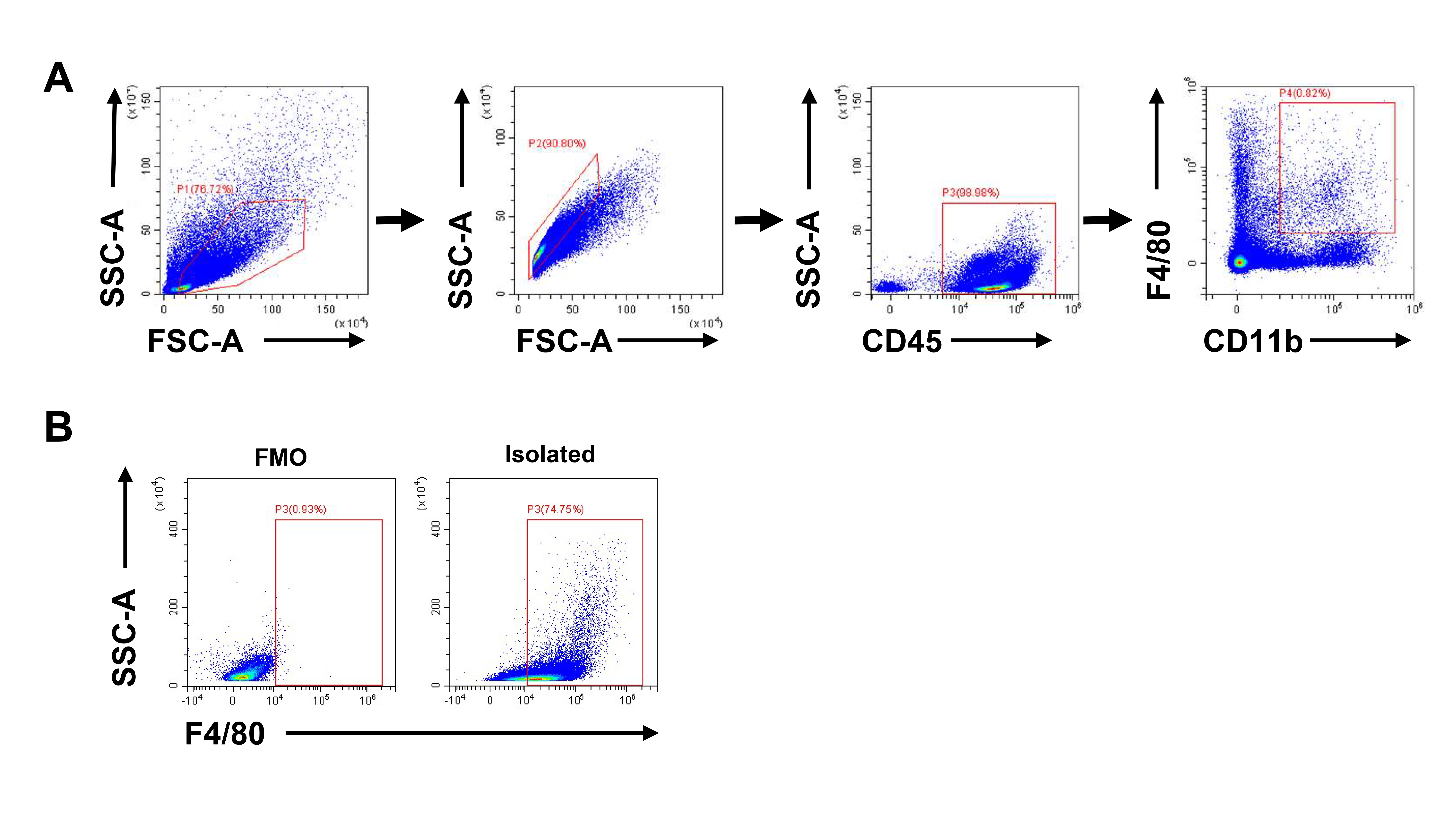
**

**Supplementary Fig. 1**

**Fig. S1A.** Gating strategy for macrophages. **Fig. S1B.** Macrophage sorting purity.


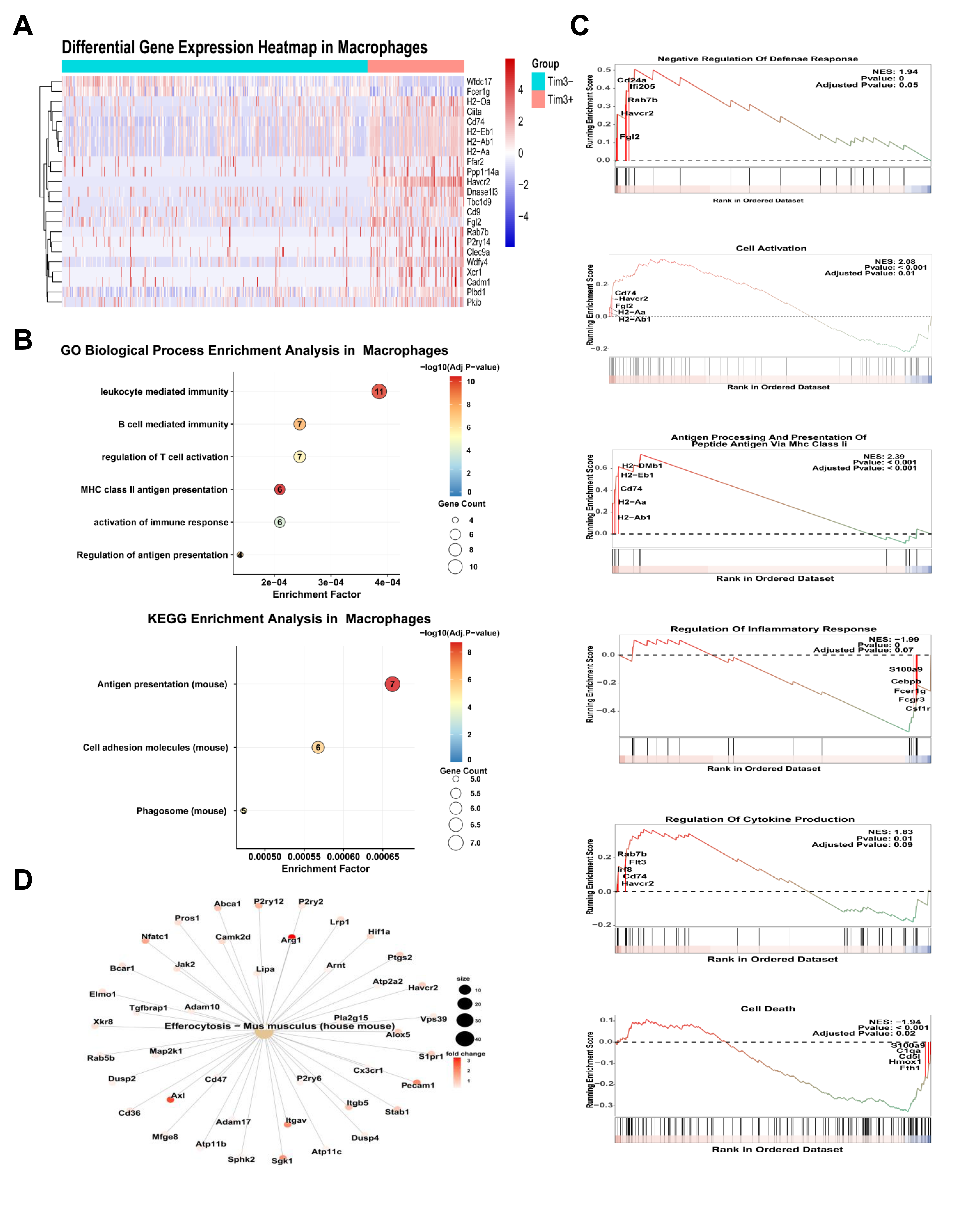


**Supplementary Fig. 2**

**Fig. S2A.** The heatmap based on the genes in Supplementary Table 2 visually presents the gene expression differences between the two groups of macrophages. **Fig. S2B.** The GO and KEGG enrichment analysis bubble chart presents the enriched pathways of Tim-3^+^ and Tim-3^-^ macrophages. **Fig. S2C.** The relaxed differential genes in the GSEA enrichment score plot illustrate the functions of Tim-3^+^ and Tim-3^-^. **Fig. S2D.** The cnetplot illustrates the correlation between the efferocytosis effect in macrophages and *Havcr2*.

**
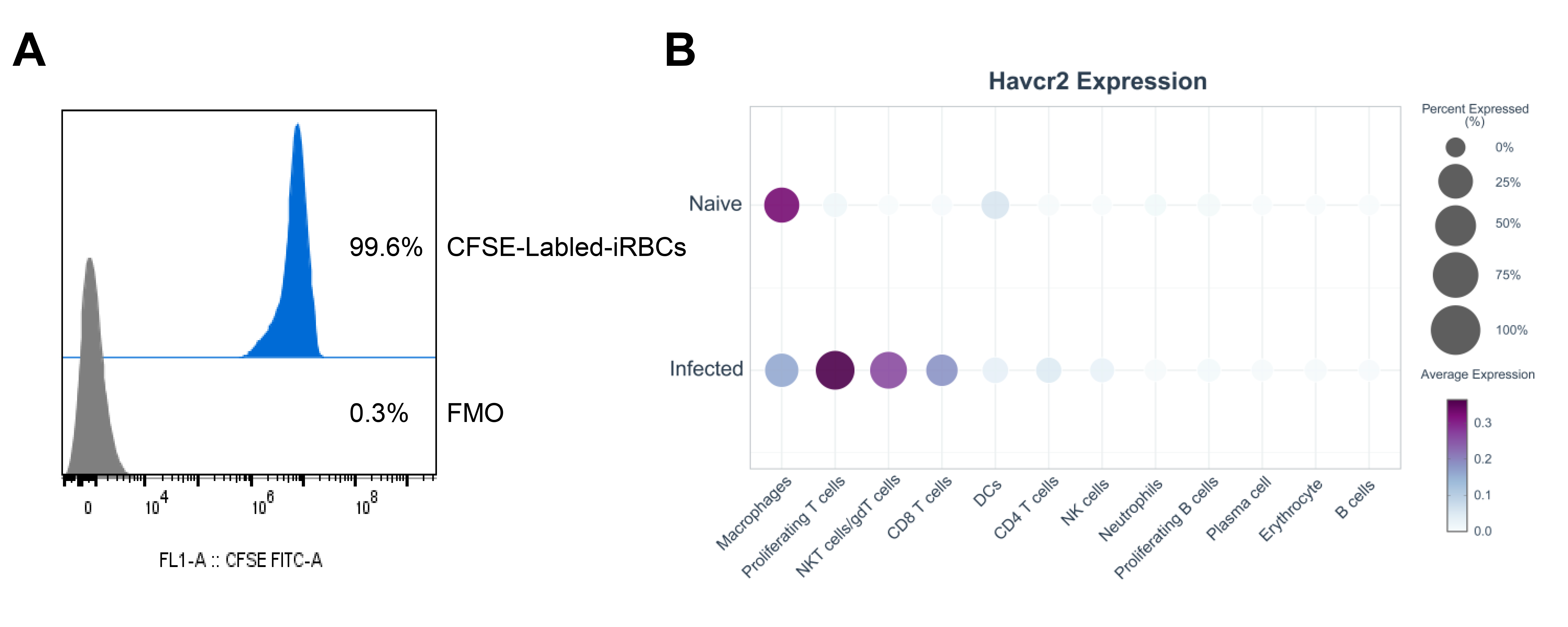
Supplementary Fig. 3**

**Fig. S3A.** CFSE-labeled iRBCs. **Fig. S3B.** Average *Havcr2* expression in splenic immune cell from naïve and infected mice.
